# Culture, politics and being more equal than others in COVID-19: some psychological anthropology perspectives

**DOI:** 10.1177/00812463211012646

**Published:** 2021-06

**Authors:** Indira Pillay

**Affiliations:** Masters student, University of Pretoria, South Africa

**Keywords:** Anthropology, community, COVID-19, culture, pandemic, psychology

## Abstract

The COVID-19 pandemic changed how we view the world, human behaviour, and societal structures and institutions. The emerging subdiscipline of psychological anthropology is well placed to provide a perspective on the way individuals and communities are affected by and respond to the pandemic, as well as the fallout from government responses and prevention strategies. Moreover, this viewpoint enables insights into the workings of societal structures and agents of power in the context of a health crisis that is worsened by poverty, inequality, and structural violence. How communities respond and adapt to the ‘new normal’ are critical to holding governing structures accountable in situations where poor leadership, mismanagement, and unethical behaviour have been evident.

The COVID-19 pandemic disrupted our lives, with calamitous effects on the economic, social, and health status of individuals worldwide. The pandemic has made it necessary to wear masks, frequently sanitise hands, and avoid close contact with others. Fear and panic spread globally. By September 2020, there were close to 30 million cases and almost 1 million deaths worldwide (Worldometer, 2020), with many issues coming to light about how the world was affected by the virus, and not just in terms of the disease process.

The COVID-19 pandemic demanded changes in human behaviour and community life. In other words, attention needed to be given to areas of psychological and anthropological concern. These two fields of study share much in terms of their focus on humans in their individual and community contexts. The links between psychology and anthropology go far back, considering the influential works of anthropologists like Margaret Mead, John, and Beatrice Whiting, among others, in developmental psychology. Psychological anthropology, which developed as a subfield of anthropology, refers to the study of human behaviour, development, and experience in the context of the ideologies and institutions of the sociocultural environment (LeVine, 2010).

Anthropology historically concerned itself with communities and how they evolve and adapt to prevailing contexts and challenges. An example of society’s adjustment to an epidemic recently came to light in Florence, Italy, where the plague caused thousands of deaths during the 17th century. Not being allowed to serve customers in the usual manner, wine producers carved small holes in the walls or doors of their houses, known as *buchette del vino*, so they could pass flasks of wine through, without touching customers (Squires, 2020). The coin payments, passed on a metal tray, were sterilised in vinegar to prevent infection. These *wine windows*, which were sealed for centuries, are being re-opened during COVID-19 to sell drinks in a safer manner – an indication of human resilience and coping in challenging circumstances.

It is against this background of human challenges and adaptation that I explore some issues relating to the COVID-19 pandemic and its interrelationships with human behaviour, communities, and societal institutions.

## Community, culture, and COVID-19

In pandemics like COVID-19, the critical prevention responsibilities lie in the behaviours of individuals and communities. The World Health Organization (WHO) (2020) emphasised the role of community behaviour in reducing viral spread, advising the public to ‘Make sure you, and the people around you, follow good respiratory hygiene. This means covering your mouth and nose with your bent elbow or tissue when you cough or sneeze’ (webpage). This illustrates the interdependence among individuals for their wellbeing.

The well-known African proverb, ‘Umuntu ngumuntu ngabantu’, which means ‘I am because we are’, helps us understand that the community is bigger than its individuals. One’s actions feed into the community’s purpose, and the community gives meaning to the individual. Consider then, that the foremost idea in COVID-19 prevention is wearing a mask, the idea being that ‘I am wearing a mask to protect you’. This is in harmony with the philosophy of Ubuntu, emphasising the interdependence of human beings in the achievement of wellbeing and community progress.

The pandemic highlights the value of collectivist cultures over individualist cultures. Years ago, cross-cultural psychologist Harry Triandis (1988) pointed to the major differences between individual and collectivist cultures. Individualists are driven by personal goals geared towards attaining personal aims, while collectivists develop their goals according to the broader needs of their communities. This means thinking unselfishly and behaving altruistically to benefit society. A recent study of young adults advocated promoting cultural orientations of shared common goals and interdependence as important strategies in reducing the COVID-19 spread and psychological maladjustment (Germani et al., 2020). Public health approaches are aimed at population-based utilitarian value, and the willingness to act for the good of society is more evident in collectivist communities (Habersaat et al., 2020). The Ubuntu philosophy and other traditional African and Eastern ways of life have much to contribute to enhancing approaches to pandemics like COVID-19. Steve Biko (1978) noted ‘. . . one can extract from our indigenous cultures a lot of positive virtues which should teach the Westerner a lesson or two. The oneness of community for instance is at the heart of our culture’ (p. 30).

COVID-19 changed daily lives, and an important community practice came to a halt. Visiting family and friends was curtailed by the lockdown, which involved government regulations restricting movement and social gatherings to ensure people remain in their homes, thereby reducing viral transmission. This, unfortunately, took away the sense of community and togetherness that is an essential human need. Caring for the elderly, for example, changed due to their greater vulnerability to severe illness. Groceries and medication were left on their doorstep to reduce physical contact. However, while separating the elderly to independent or institutional living is uncharacteristic of African societies, most experience severe economic hardship with many supporting extended family on their state pensions (Rupiya, 2020). For the elderly in nursing homes and frail care facilities, those spaces turned out to be rampant breeding grounds for the virus with numerous deaths worldwide. In South Africa, people above 60 years accounted for more than half of COVID-19 deaths, and this vulnerability resulted in a curtailment on family visits, increasing their fear, loneliness, and desolation (Smit, 2020). This should not happen to society’s elders. Psychiatrist and medical anthropologist, Arthur Kleinman, noted that the inequitable impact on society’s most vulnerable individuals revealed the brokenness of society (Miller, 2020).

It is clear that COVID-19 has proven to be more than simply a disease entity, considering its enormous social impact. There is, therefore, a need for pandemic interventions and public health strategies to be developed in collaboration with social scientists, rather than based solely on biomedicine. From an anthropological perspective, McNeil (2020) noted that the discipline has a considerable history of trying to make sense of how societies respond to health crises, citing the examples of Ebola and HIV/AIDS where it has been shown that medical interventions alone are insufficient. Speaking at the recent Harvard – Weatherhead Forum on COVID-19, Allan Brandt (2020) lamented the insufficient involvement of social scientists in national response planning initiatives, while Vikram Patel (2020) asserted that ‘biomedicine has found its light in the sun and dug its heels deep into the ground’. Undervaluing the role of the social sciences in viral pandemics is short-sighted as it ignores the crucial human element with its social and cultural complexities. Disciplines like anthropology and psychology have much to contribute, given their understanding of individuals, communities, and human behaviour.

A significant effect of the pandemic has been the way it deprived the community of *community*. Fuentes (2020) noted that ‘Humans evolved as beings whose needs to touch and be touched, to converse, debate and laugh together, to smile and flirt with one another and to interact in groups are central to healthy lives’ (p. 24). The lockdown effort to control the virus necessitated physical distancing and prohibition on social gatherings, which runs contrary to human behaviour and the way the species evolved. Coinciding with evolutionary development, the past century saw human life become more connected across the planet’s physical and social spaces. Although a positive feature, this connectedness, through easy travel across continents, facilitated the rapid viral spread through the global community.

Another evolutionary development, namely information and communication technology, helped individuals and communities cope with the virus and lockdown, through easy information access and social contact. Both are crucial in dealing with the pandemic. Despite the bad press that social media sometimes gets, there has been some benefits. Das and Ahmed (2020) argue that social media played a central role in disseminating vital information about the pandemic, noting for example, that the spread of the virus across Italy became known earlier on social media than on mainstream media. In disaster situations, early warning systems, provided they are legitimate, have great value and can save lives. A further benefit of social media lies in its messaging platforms that allow friends and family to keep in frequent contact inexpensively. This has been important, given the physical distancing requirement and the need for people to monitor the wellbeing of significant others. Perhaps this is where the commonly used term, *social distancing*, needs correcting. Physical distance is necessary to reduce viral transmission, but social distance is not a good idea because closer social contact (through social media, telephone, etc.) facilitates support, allowing people to monitor the wellbeing of significant others.

## Healing, grief, and rituals in the pandemic

COVID-19 shifted the way we approach almost every aspect of life, including education, health care, work, shopping, and social contact, among others. Religious worship, cultural rituals, death, grief, and mourning are among the life practices affected. However, unlike other aspects of life that changed substantially since the days of early humans, cultural rituals and religious practices are relatively long-standing and more rooted in the psyche of human beings. Cultural rituals play a valuable role in supporting life transitions, crisis management, forging collective identity, reinforcing belonging, re-motivating purpose, and reducing anxiety (Nelson-Becker & Sangster, 2019). Rituals are so critical to family and community life that Durkheim and Cosman (2001) viewed them as the glue that holds society together.

In modern society, some cultures have a greater array of rituals, but COVID-19 prohibited many rituals due to the social and behavioural constraints. Funerals, for example, had to be substantially modified to comply with COVID-19 regulations. Not only were mourner numbers restricted, but open caskets were disallowed, and some cultural rites could not be performed. Usually, death brings together family, relatives, and friends who give much needed support to the bereaved, but this was not possible in the pandemic, or it happened in a diluted form.

When a person passes on, especially in collectivist cultures, as in African and Eastern traditions, many rituals are necessary. These have meaning to families and communities, as they are believed to help the soul of the individual in the afterlife, while also helping the bereaved family. The ceremonial duties and rituals to be performed by family members have to be abbreviated, rushed, or curtailed – but how are these forced ‘short-cuts’ impacting the bereaved? Psychologists, helping individuals and families with grief, have to take note, and understand that the missed rituals may affect the coping of grief-stricken individuals. Anthropologist Bronislaw Malinowski (2018) noted that historically death was considered as much more than the departure of a family member, and that the ceremonial rituals addressed the fears, sadness, and disheartenment faced by the family, serving to unite and strengthen them through their time of grief. Reports of families’ inability to grieve in the usual, unhurried, ceremonial manner have been frequent due to COVID-19 (Haffejee, 2020).

A recent mix-up at a Pietermaritzburg hospital due to prohibition on viewing the deceased has caused serious anguish to two families of different religions. The first family performed cultural rituals, cremation, and scattering of ashes into the sea, in accordance with their religious beliefs. However, the second family insisted on viewing the deceased and discovered it was not their family member. Investigations revealed that the cremated body belonged to the second family whose religious practices do not permit cremation and scattering of the ashes in the sea (Ngcobo & Bennie, 2020). Such an error has profound emotional and cultural impact on affected families. They were not able to perform the rituals that they and their loved ones believed in all of their lives. Anthropologists have described COVID-19 deaths as a very lonely experience for family members (Soloman & Buchbinder, 2020). Certainly, the emotional impacts are greater than we may realise.

The pandemic also affected various rites of passage. For example, in some cultures, a ceremonial ritual performed by elder women from family and friends marks the attainment of menarche in girls. This is considered a vital rite of passage, but cannot be done in its entirety due to physical distancing, and restricted gatherings. Given the current, forced trends, we have to wonder whether COVID-19 is pushing collectivist cultures into more individualistic ones. It is likely that the pandemic may cause evolutionary changes in our social and cultural practices that may someday be viewed as part of our culture and tradition, even though they were moulded by the pandemic. This is how culture and tradition adapt and evolve through history.

Just as governments had to balance prevention strategies with the necessary restarting of the economy, schooling, and civil services, they must seek to balance prevention strategies with cultural practices that bring meaning to the lives of individuals, families, and communities. Geertz (1973) argued, ‘there is no such thing as a human nature independent of culture’ (p. 11). Although lockdown strategies are considered life preservation strategies, the value of life without meaning is also subject to question. Life transitions within the developmental cycle are critical milestones and their cultural recognition and marking through traditional rituals impact significantly human and family wellbeing. This is not surprising, considering that culture influences every aspect of human life (Swartz, 1998).

## Leadership during COVID-19

Pandemics, by definition, require a coordinated global response (Bowman, 2020), but while most world leaders took COVID-19 seriously, a few did not. The US President Donald Trump believed it was similar to the ordinary flu, and delayed appropriate response. Many believe his administration’s slow response was responsible for thousands of preventable deaths and that he should be held to account, even suggesting criminal liability (Mystal, 2020).

In an attempt to deflect attention from his failings, Trump blamed China for the virus, referring to it as the ‘Chinese virus’, even blaming the WHO, his political opponents, and the Black Lives Matter campaign for the viral spread. His response of withdrawing funding to the WHO seemed childish and irresponsible. This type of response serves only to perpetuate the kind of poorly informed ‘Fearbola’ that developed as an irrational fear and prejudiced response to the Ebola outbreak in Africa which was reminiscent of the initial US response to HIV/AIDS (Petrow, 2014). A shift in US policy and pledge to continue involvement with the WHO following the inauguration of Joe Biden as President reflects the wider concerns about Trump’s leadership (Nebehay, 2021).

Africa also gained attention for poor COVID-19 leadership. Madagascan President Andry Rajoelina pushed the idea of an herbal COVID-19 cure that most of the world did not take seriously. Although four African country leaders reportedly ordered supplies of Madagascar’s ‘cure’, most leaders at the African Union meeting in April showed no support (Adeoye & Allison, 2020). South Africa went through a similar situation when an earlier Minister of Health made claims about garlic and beetroot delaying the progression of HIV to AIDS, instead of rapidly initiating an antiretroviral (ARV) treatment programme. Harvard research subsequently revealed that more than 330,000 lives were lost because of delayed ARV treatment, and 35,000 children were born with HIV because mother-to-child transmission prevention programmes were not implemented (Chigwedere et al., 2008). Years ago, sociologist C Wright Mills (1956) noted that forces beyond their control drive the lives of ordinary citizens, but argued, nevertheless, that citizens need to hold agents of power and control accountable for the ills they cause in society. We need to realise that simply identifying the troubles caused by the *power elites* is not good enough.

Although it is expected that COVID-19 would put health systems in poor countries under severe strain, reports from some high-income countries are interesting. For example, Carlson (2020) highlighted the tragic undersupply of health services to Native Americans, noting only 625 beds, six intensive care beds, and 10 ventilators for more than 2.5 million indigenous Americans. The *Doctors without Borders* relief organisation that provides health services to poor countries assisted indigenous communities in the United States (Kauffman, 2020), while Donald Trump constantly asserted that the United States was doing well in its fight against the pandemic. Are Native Americans not part of the United States and why did we not hear him speak much about their COVID-19 challenges?

In Canada, Human Rights Watch raised concern about the health of indigenous communities due to systemic inequalities and discrimination, arguing that they may suffer disproportionately in the COVID-19 pandemic (Carling & Mankani, 2020). According to Seymour (2020),

Canada’s Minster of Indigenous Services, Marc Miller said social determinants of health, such as unsafe drinking water, crowded housing, lack of health professionals, poor infrastructure and chronic diseases, play a role in making Indigenous communities more vulnerable to the coronavirus. (webpage)

Similar concerns have been raised about Australia’s indigenous communities and their marginalisation. Yashadhana et al. (2020) argued that ‘The political, social and cultural determinants of health are stacked against Indigenous Australians and Indigenous peoples globally; this multiplies the risks to, and vulnerabilities of these communities to infection and mortality from COVID-19’. (p. 2).

The Western world’s responsiveness to the health and social conditions of indigenous inhabitants has been appalling. Considering the histories of colonisation and the ruthless manner in which Westerners shifted indigenous inhabitants to the fringes of society, with inadequate basic services and increased health vulnerabilities, it is long overdue that wealthy nations come to grips with what they have done and make reparation. Health and humanitarian crises such as the COVID-19 pandemic are likely to devastate these communities. The irony is that these indigenous communities are in wealthy countries. These are examples of *structural violence* that Johan Galtung (1969) referred to when communities are deprived of their basic needs by the prevailing social and political structures. Structural violence includes the economic and political arrangements that organise the social world such that it puts people in harm’s way (Farmer et al., 2006).

With few exceptions, clinicians are not trained to understand such social forces, nor are we trained to alter them. Yet it has long been clear that many medical and public health interventions will fail if we are unable to understand the social determinants of disease. (Farmer et al., 2006, p. 1686)

## The cash cow named COVID-19

Throughout the world, governments have released extra funds to finance relief efforts during the pandemic, considering the loss of income due to lockdown measures and other costs associated with the pandemic. The South African government borrowed billions from the International Monetary Fund, in addition to its initial relief package of R500 billion. However, for some, the pandemic brought new opportunities for devious self-enrichment.

The Auditor-General South Africa (2020) released a report noting fraudulent activities in the procurement of personal protective equipment (PPE) and even deficient quality PPE purchased for some schools. The Auditor-General also found conflicts of interest in the awarding of PPE supply contracts, prices inflated up to 5 times the prescribed price and contracts awarded to suppliers with no history of PPE provision. In addition, it was discovered that payments were made in terms of the Temporary Employee/Employer Relief scheme to individuals recorded as deceased, under the legal employment age, working for government, social grant recipients, and state-funded students. At more local levels, Corruption Watch (2020) reported that government officials stole food parcels meant for the poor. However, this is not just a South African problem.

A recent survey across 58 countries, comprising 76% of the global population, found that 81% of the countries experienced PPE-related fraud, 62% had an illicit market for the products, and 58% reported embezzlement of health care funds (Milata, 2020). In the United Kingdom, a £252.5 million PPE contract awarded to an investment company raised eyebrows, and similar PPE contracts were awarded to a sweets manufacturer, a recruitment agency, and a pest control product company (Coburg, 2020).

Canadian Prime Minister Justin Trudeau was also implicated in corrupt dealings relating to COVID-19 relief funds. During his 5 years in office, he was already found in breach of his country’s conflict of interest regulations twice, before the latest ‘political scandal over his government’s decision to award a hefty no-bid contract to a charity that has ties with his family’ (Porter, 2021, webpage). It was revealed that the charity was awarded an emergency youth volunteer programme government contract, worth hundreds of millions of dollars, and that Trudeau’s mother and brother received in excess of US$200,000 from the organisation over the past few years for speaking engagements.

The extent to which governments and leadership are speaking out against corrupt practices and the widespread theft of public funds is troubling. Responses from some leaders have been mild and less critical or action-oriented than people would like. Considering taxpayers’ anger about the squandering of their hard-earned money, there is a public expectation of stronger responses from leaders. It calls for urgent activism against corruption, to speak truth to power, similar to the effective uprising by young people against apartheid and for Black Lives. The looting of COVID-19 relief funds meant to alleviate poverty and keep people safe demands louder voices from its leaders.

We can consider here Michel Foucault’s (1983) idea of frankness or *parrhesia*, which he described as the act of courageously speaking the truth, without attempting to hide any part of what is being criticised. Moreover, he saw personal risk and even danger as an integral part of this outspokenness because the truth may ruffle some feathers. Even though silence is an option, the truth must be spoken as a duty to the self and others. It is, in fact, an act of freedom. Those in power have the moral and ethical duty to locate and act on the injustices facing their citizens. That is why they were elected to the positions they hold. Commenting on Foucault’s *parrhesia*, Dyrberg (2016) added that ‘Power concerns the ability and the audacity to face up to important political tasks and to take action at the right time authoritatively and with resolve’ (p. 265).

## Some are more equal than others

The pandemic brought out the best in many individuals, communities, and organisations, but it also brought out the worst in some, especially those who should have modelled high levels of moral and ethical behaviour. In the celebrity context, social media was abuzz with the rantings of Canadian music icon Bryan Adams when his concerts were cancelled due to the pandemic. His statement criticising Chinese eating and cultural practices, and blaming them for the pandemic, was regarded as racist and xenophobic (Savage, 2020). However, another problem with Adams’ behaviour is his apparent self-centredness, or what psychology terms *egocentricism*. Millions, and possibly billions, of people globally have been pushed into starvation due to the pandemic, but his greater concern seemed related to the cancellation of his concerts due to COVID-19.

However, politicians stole the show. A day after the start of the COVID-19 lockdown in South Africa, we saw pictures of Cabinet Minister Stella Ndabeni-Abrahams socialising with friends, which she later explained as a ‘meeting’. The R1000.00 fine and 2-month special leave imposed on her was a slap on the wrist, while other citizens were harassed and beaten by police for breaching lockdown regulations, and some died as a result. According to anthropologist, Laura Nader (1974), ‘studying up’ is crucial to social change because researchers focus more on the society’s less fortunate while not putting the spotlight sufficiently on agents of power and control. Without focusing on those in high positions, we do not get that data we need to inform the changes necessary to promote democracy.

Donald Trump’s refusal to wear a mask when most of the world did so is an example of George Orwell’s *Animal Farm* character Napoleon, arguing that some beings are more equal than others. According to Wilkinson (2020), wearing a mask is not only to prevent germs entering your body but it is also a ‘civic responsibility’. At the start of the lockdown, wearing a mask was put forward with the idea being ‘I am wearing a mask to protect you’. Therefore, is a person who refuses to wear a mask saying ‘I don’t care’ about the health of others around me?

Some world leaders and high-ranking officials have also disrespected lockdown regulations in their countries. This included the travels of Britain’s most senior Brexit official, Dominic Cummings, resulting in calls for his resignation. The Czech Prime Minister was criticised for going for a walk for an ice cream with the country’s chief epidemiologist, and for being at his hairdresser without a mask, despite it being mandatory in closed spaces (Morgan, 2020). Brazil’s President Jair Bolsonaro criticised lockdown measures in his own country despite high case numbers. He told journalists he planned to attend a barbecue party that was later cancelled, but was seen jet skiing at a lake (Sims, 2020).

Power politics have been evident in the way leaders and government officials are left to regulate their own behaviour, but the rest of society is subjected to vigorous monitoring and policing to impose social control. The message being that government officials are capable of self-control and righteousness, which the rest of society is not, and therefore, must be watched. We can think here of Foucault’s (1977) *panopticism*, the idea of individuals developing an internal monitoring system to regulate their behaviour without needing to be watched. To a large extent, the pandemic lockdown produced a *panopticon*, where the vast majority of citizens regulated their behaviour to conform to the recommended prevention strategies (Human Sciences Research Council, 2020). The irony is that not all those in positions of influence or authority regulated their behaviour, or displayed moral and ethical conduct.

The issue of moral and ethical conduct in the global context is evident in the problem of *vaccine nationalism*, which refers to selfish attempts by governments and leaders to acquire excessive quantities of vaccines for their countries in a way that depletes stocks and limits availability to other (especially poorer) countries. For example, Canada, which has a population of 38 million, secured 362 million doses by the end of January 2021, while the United Kingdom secured 400 million doses which is about 6 times greater than the needs of its population (Lock, 2021). In a webinar on Al Jazeera (2021) around vaccine nationalism, Max Lawson, the Head of Inequality Policy at Oxfam International, emphasised that taxpayers’ money funded pharmaceutical companies’ vaccine development. Therefore, pharmaceutical companies’ interests in intellectual property and profit-making are inappropriate.

## Conclusion

Community life is at the centre of the pandemic, and certain changes were necessary to reduce the viral spread. However, some of the changes affect long-standing cultural practices and daily living. It is critical that focus is not only on avoiding the virus but also on recognising that there is life after COVID-19, and people need to continue with the belief systems that gave them meaning in the past. The pandemic also highlighted social inequality and structural violence that need addressing by agents of power who must be held to account for faulty leadership, allowing some to steal and feel ‘more equal than others’, while millions face poverty and hardship.

One of the lessons we learned from COVID-19 is that pandemics are a part of our future. The precise role played by humans in unleashing viruses is not completely understood, but our behaviour and misuse of the natural environment are implicated. Climate change is considered a key driver of the global increase in infectious diseases (Flahault et al., 2016). Although history shows that humans have the capacity to adapt and overcome such challenges, we have not evolved to the point of respecting our planet, and we need to achieve that quickly if we are to continue as a species that is healthy and deserving of the fruits of our environment.
